# Evolutionary Plasticity in Detoxification Gene Modules: The Preservation and Loss of the Pregnane X Receptor in Chondrichthyes Lineages

**DOI:** 10.3390/ijms20092331

**Published:** 2019-05-10

**Authors:** Elza S. S. Fonseca, Raquel Ruivo, André M. Machado, Francisca Conrado, Boon-Hui Tay, Byrappa Venkatesh, Miguel M. Santos, L. Filipe C. Castro

**Affiliations:** 1 CIIMAR/CIMAR—Interdisciplinary Centre of Marine and Environmental Research, 4450-208 Matosinhos, Portugal; fonseca.ess@gmail.com (E.S.S.F.); ruivo.raquel@gmail.com (R.R.); andre.machado@ciimar.up.pt (A.M.M.); fi.silva@campus.fct.unl.pt (F.C.); santosmafm@gmail.com (M.M.S.); 2 FCUP—Faculty of Sciences, Department of Biology, University of Porto, 4150-177 Porto, Portugal; 3 Comparative Genomics Laboratory, Institute of Molecular and Cell Biology, A*STAR (Agency for Science, Technology and Research), Biopolis, Singapore 138673, Singapore; mcblab46@imcb.a-star.edu.sg (B.-H.T.); mcbbv@imcb.a-star.edu.sg (B.V.)

**Keywords:** nuclear receptors, gene loss, detoxification, endocrine disruption

## Abstract

To appraise how evolutionary processes, such as gene duplication and loss, influence an organism’s xenobiotic sensitivity is a critical question in toxicology. Of particular importance are gene families involved in the mediation of detoxification responses, such as members of the nuclear receptor subfamily 1 group I (NR1I), the pregnane X receptor (*PXR*), and the constitutive androstane receptor (*CAR*). While documented in multiple vertebrate genomes, *PXR* and *CAR* display an intriguing gene distribution. *PXR* is absent in birds and reptiles, while *CAR* shows a tetrapod-specific occurrence. More elusive is the presence of *PXR* and *CAR* gene orthologs in early branching and ecologically-important Chondrichthyes (chimaeras, sharks and rays). Therefore, we investigated various genome projects and use them to provide the first identification and functional characterization of a Chondrichthyan PXR from the chimaera elephant shark (*Callorhinchus milii*, Holocephali). Additionally, we substantiate the targeted *PXR* gene loss in Elasmobranchii (sharks and rays). Compared to other vertebrate groups, the chimaera *PXR* ortholog displays a diverse expression pattern (skin and gills) and a unique activation profile by classical xenobiotic ligands. Our findings provide insights into the molecular landscape of detoxification mechanisms and suggest lineage-specific adaptations in response to xenobiotics in gnathostome evolution.

## 1. Introduction

Nuclear receptors (NRs) are central constituents of animal endocrine systems. These ligand-dependent sensors act as transcription factors, regulating key physiological processes including metabolism, development, reproduction and nutrient utilization [1]. Importantly, NRs are also directly exploited by xenobiotics, causing numerous examples of physiological impairment (e.g., [2,3]). Two critical components of the vertebrate’s “chemical defensome” are the pregnane X receptor (*PXR*) and the constitutive androstane receptor (*CAR*) [4]. These are part of nuclear receptor subfamily 1 group I (NR1I), which also includes the vitamin D receptor (VDR). *PXR* and *CAR* were originally identified as xenobiotic sensors, since they regulate genes involved in drug metabolism such as phase I cytochrome P450 (e.g., CYP3A4, CYP2B6 and CYP2C), phase II transferases (e.g., uridine 5′-diphospho(UDP)-glucuronosyl transferase and glutathione-*S*-transferase), and drug transporters. Moreover, *PXR* is notoriously involved in other metabolic processes including energy homeostasis, inflammatory responses, cell proliferation, apoptosis, and tumour development [5,6,7]. The taxonomic distribution of *VDR/PXR/CAR* gene orthologs is remarkably mutable [8,9,10,11]. In vertebrate species, *VDR* is found in both cyclostomes (lampreys) and gnathostomes [12]; *CAR* occurs in tetrapods [10,13]; while *PXR* genes have been described and characterized in amphibians [10] and mammals [10]. On the other hand, teleost genomes, such as zebrafish (*Danio rerio*), also retain *PXR*, but this is not an universal condition found throughout teleost lineages [10,11], consistent with the highly derived nature of their genomes [14]. Importantly, synteny supports the hypothesis that the absence of *PXR* in birds, reptiles, and some teleosts, as well as *CAR* in ray-finned fish, is due to secondary gene loss [10]. Genome comparisons between human and teleost *PXR*, *CAR*, and *VDR* orthologous genomic regions further implicates whole genome duplications (2R) as the underlying cause of the *NR1I* gene expansion [10,15]. These observations suggest that *VDR*, *PXR*, and *CAR* first appeared in the ancestors of vertebrates and should be present in early lineage genomes such as Chondrichthyes (cartilaginous fish). Consistently, *VDR* has been described and functionally characterized in cartilaginous fishes [12]. *PXR* and *CAR* orthologs have not been described in Chondrichthyes, although the presence of the former has been suggested [10]. Here we thoroughly investigate the gene repertoire of the NR1I subfamily, central components of the “chemical defensome”, in Chondrichthyes. Cartilaginous fishes are divided into two branches: Holocephali (chimaeras) and Elasmobranchii (sharks, rays and skate). Together, they are a highly diversified group of early branching vertebrates, representing important components of aquatic ecosystems and food webs, and thus are key ecological indicators [16,17].

## 2. Results

### 2.1. Identification of nuclear receptor subfamily 1 group I (NR1I) Ortholog Genes in Chondrichthyes

**Figure 1:**
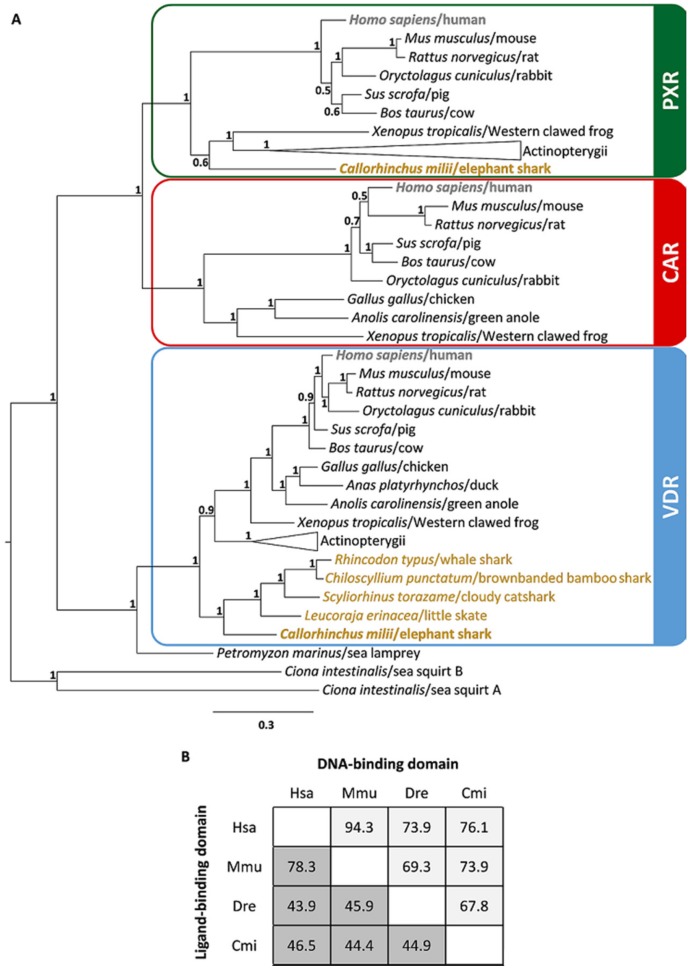
The *NR1I* gene repertoire in Chondrichthyes. (**A**) Bayesian phylogenetic tree of vitamin D receptor (*VDR*), pregnane X receptor (*PXR*) and constitutive androstane receptor (*CAR*) genes. The numbers at the nodes represent the statistical support expressed in Bayesian posterior probabilities. Actinopterygii are represented by *Danio rerio* (zebrafish), *Cyprinus carpio* (European carp), *Oryzias latipes* (medaka), *Oreochromis niloticus* (Nile tilapia), *Scleropages formosus* (Asian arowana), and *Lepisosteus oculatus* (spotted gar). Chondrichthyes are highlighted in yellow: Elasmobranchii (little skate, brownbanded bambooshark, whale shark and cloudy catshark) and Holocephali (elephant shark) in bold. (**B**) Percentage of amino acids, identity of DNA, and ligand binding domains between human (Hsa), mouse (Mmu), zebrafish (Dre), and elephant shark (Cmi) PXRs.

To determine the gene complement of *VDR/PXR/CAR*-like genes in Chondrichthyes species, we examined five genome datasets from two subclasses: Holocephali (chimaeras) and Elasmobranchii (sharks and rays) [18,19,20,21,22]. Our search identified one or two genes with similarities to *VDR/PXR/CAR* genes in the elephant shark (*Callorrhyncus milii*), little skate (*Leucoraja erinacea*), cloudy catshark (*Scyliorhinus torazame*), brownbanded bamboo shark (*Chiloscyllium punctatum*), and whale shark (*Rhyncodon typus*). To establish the orthology of the retrieved sequences, we performed phylogenetic analysis (Figure 1A), including the described *VDR/PXR/CAR* gene sequences from mammals, birds, reptiles, amphibians, teleosts, lepisosteiformes, cyclostomes, and tunicates (Table S1). Three statistically-supported sequence clades were retrieved, consistent with the separation into *VDR*, *PXR* and *CAR* genes (Figure 1A). We then compared the two critical functional domains of the *Cmi*PXR: the DNA-binding domain (DBD) and the ligand binding domain (LBD). *Cmi*PXR shares around 70% of its sequence identity to other PXR-DBDs (Figure 1B), with the LBD identity values displaying significantly lower values (Figure 1B).

### 2.2. Synteny Analysis of NR1I Ortholog Genes

**Figure 2:**
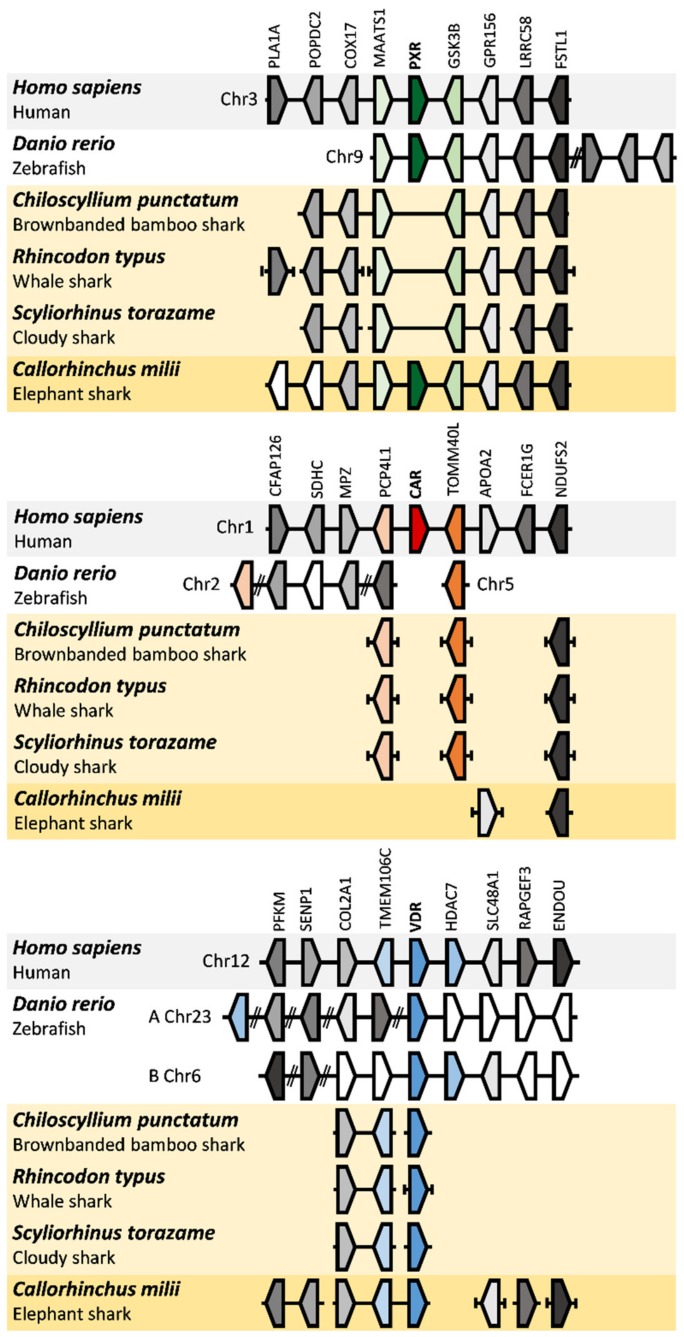
Schematic representation of syntenic pregnane X receptor (*PXR*), constitutive androstane receptor (*CAR*), and vitamin D receptor (*VDR*) regions. Human, zebrafish, brownbanded bamboo shark, whale shark, cloudy catshark, and elephant shark genomic locations of *VDR*, *PXR*, and *CAR* genes. The genomic locations of human *PXR*, *CAR*, and *VDR* were used as reference and highlighted in grey. The double slashes in zebrafish chromosomes symbolize discontinuity in the chromosome representation. The scaffolds with chondrichthyan orthologs were highlighted in yellow (dark yellow for elephant shark).

To further verify the orthology of these novel gene sequences and to discriminate between true gene loss or absence of sequencing data, we next verified the genomic location of *VDR*, *PXR*, and *CAR* within the syntenic locations in the genomes of the elephant shark, cloudy catshark, brownbanded bamboo shark, and whale shark, using the human and zebrafish gene loci composition as reference, as shown in Figure 2. The scattered assembly and small contiguous size of the current little skate genome (LER_WGS_1—GCA_000238235.1) impeded a consistent comparative analysis at this stage. The *PXR* gene from the elephant shark is flanked by *MAATS1* and *GSK3B* genes (scaffold NW_006890095.1 3.95Mb), and the overall locus composition is similar to that of other vertebrate species (Figure 2). In the Elasmobranchii species analysed here, and despite the global synteny conservation, no intervening *PXR*-like sequence was found between *MAATS1* and *GSK3B* (brownbanded bamboo shark—scaffold scf_chipu00000056; whale shark—scaffold scf_rhity00002454; cloudy catshark—scaffold scf_scyto00010339) (Figure 2, Tables S2–S4). In the case of the *VDR* locus, we searched the scaffolds containing the human *VDR* flanking genes *TMEM106C* and *HDAC7*, but no *HDAC7* ortholog was found in any of the Chondrichthyes genomes. In the elephant shark, *VDR* was found in the same scaffold as *TMEM106C* (scaffold NW_006890370.1 105.7kb), contrary to Elasmobranchii *VDR*s (brownbanded bamboo shark—scaffold scf_chipu00001415 28.2kb; whale shark—scaffold NW_018047310.1 2.9kb; cloudy catshark—scaffolds scf_scyto00007144 and scf_scyto00012969), which were found on different scaffolds than the *TMEM106C* orthologs (brownbanded bamboo shark—scaffold scf_chipu00001599; whale shark—scaffold NW_018032445.1; cloudy catshark—scaffold scf_scyto00007676). This was probably due to missing sequencing data for the intervening genomic region (Figure 2, Tables S2–S4). In Chondrichthyes, the *CAR* locus is dispersed in comparison to humans (Figure 2, Tables S2–S4).

### 2.3. Gene Expression Analysis of the Elephant Shark Pregnane X Receptor (PXR)

**Figure 3:**
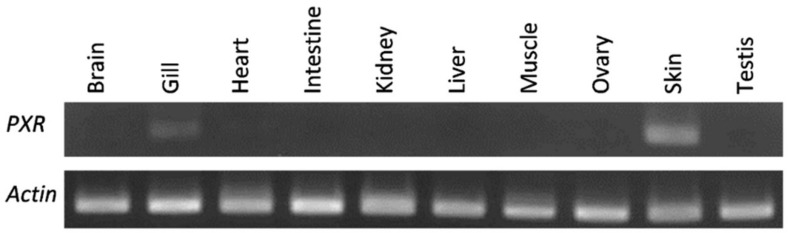
*PXR* expression pattern on an elephant shark tissue panel.

Next, we investigated the gene expression profile of the *Cmi*PXR in a tissue panel. Our analysis indicated that the elephant shark gene ortholog displayed a unique pattern, with restricted expression in the skin and gills (Figure 3).

### 2.4. Transactivation Assays of CmiPXR

**Figure 4:**
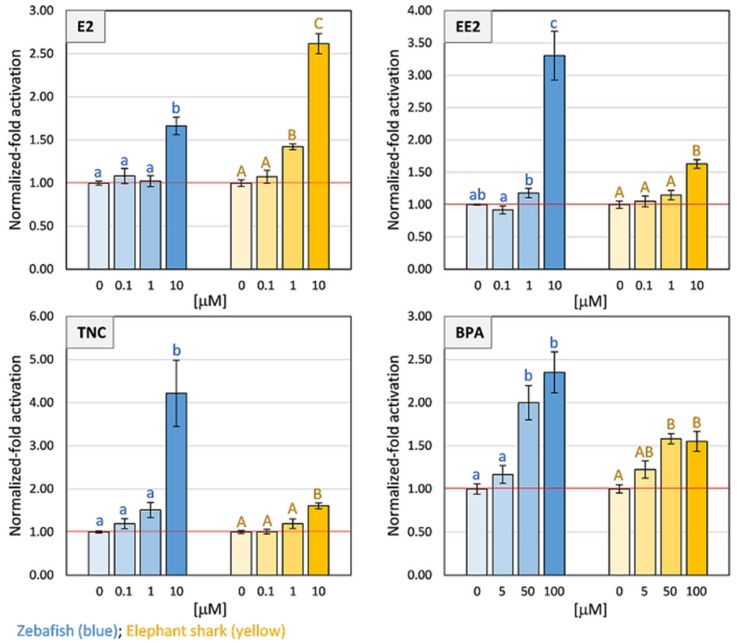
Transactivation activity of luciferase reporter gene performed in COS-1 cells mediated by the PXR ligand binding domain (LBD) pBIND constructs in the presence of 17β-estradiol (E2), 17α-ethinylestradiol (EE2), trans-nonachlor (TNC), and bisphenol A (BPA). Data represent means ± standard error of the mean (SEM) from three separate experiments (*n* = 3). The results were normalized to the control condition (DMSO without ligand). The red horizontal line represents the level of the control condition (no fold activation). Significant differences between the tested concentrations and the solvent control were inferred using one-way ANOVA. The lowercase letters (zebrafish) and the uppercase letters (elephant shark) were used to mark significant differences (*p* < 0.05).

Given the differences in the LBD sequence of *Cmi*PXR, we explored the capacity to transactivate gene expression in the presence of classical PXR ligands from different chemical categories: the natural and the synthetic steroid hormone 17β-estradiol (E2) and 17α-ethinylestradiol (EE2) respectively, and the environmental contaminants trans-nonachlor (TNC) and bisphenol A (BPA). A mammalian cell-based activation assay and the zebrafish *PXR* (*DrePXR*) were used as control. Both E2 and EE2 significantly activated (*p* < 0.05) *DrePXR* and *Cmi*PXR at high concentrations (Figure 4). Regarding the effect of the two environmental pollutants tested, both *DrePXR* and *CmiPXR* were significantly activated (*p* < 0.05) when exposed to the highest tested concentration of TNC and the two highest concentrations of BPA (Figure 4).

## 3. Discussion

By performing a comprehensive search of the *NR1I* gene repertoire in early branching gnathostome genomes, we unfolded the evolutionary history of this fundamental component of detoxification response. Notably, we were able to deduce that *PXR*, while present in the elephant shark, a Holocephali, has been most likely lost in the investigated Elasmobranchii species. The two newly-identified genes in the elephant shark fall into the *PXR* and *VDR* classes, while the single gene identified in little skate, cloudy catshark, brownbanded bamboo shark, and whale shark are bona fide *VDR* orthologs. The orthology of the new gene sequences found in this study was further confirmed by the syntenic analysis of *VDR*, *PXR*, and *CAR* locations in the genomes of the elephant shark, cloudy catshark, brownbanded bamboo shark, and whale shark. Regarding the *CAR* locus, its dispersed composition in Chondrichthyes compared to humans impedes a formal conclusion on the loss of *CAR* in these species, although this is the likeliest scenario. Additionally, gene orthologs of both *PXR* and *CAR* were not found in the two currently-available genomes of cyclostomes (sea lamprey, Pmar_germline 1.0 and Japanese lamprey, LetJap1.0). Furthermore, our analysis of amino acid identity between DBD and LBD of human, mouse, zebrafish, and elephant shark PXRs is consistent with previous studies for other species [23,24]. As expected, despite the substantial variation in sequence identity among vertebrates, we observed that the PXR–DBD is more conserved than LBD between species. This suggests that different PXRs should recognize similar response elements in the promotor of target genes, but their activation might be triggered upon binding to different ligands. The large and flexible ligand-binding pocket of PXR allows this receptor to accommodate a huge and diverse ligand range, such as endogenous ligands (5β-pregnane, progesterone, testosterone, lithocholic acids, and 17β-estradiol), antibiotics, drugs, carcinogens, and an array of environmental pollutants [25,26,27,28,29].

The occurrence of different gene complements of the NR1I subfamily in Chondrichthyes parallels similar findings in other vertebrate lineages [11,30,31]. Recently, an extensive investigation into 76 fish genomes suggests that approximately half of these species have lost *PXR* [11], in line with the description made here in cartilaginous fishes. Moreover, xenobiotic exposure experiments with classical xenobiotics, *PXR* ligands in cod (*PXR*-absent) did not show a clear transcription activation of gene coding for P450 cytochrome enzymes (*CYP3A*), as observed in mice or zebrafish [11]. In addition, promotor analysis raised the interesting possibility the *PXR*-absent species might have their *CYP3A* and *CYP1A* genes regulated by an unrelated xenobiotic-sensing transcription factor, the Aryl Hydrocarbon Receptor (*AHR*) [11]. Whether this is the case in Chondrichthyes that have lost *PXR* remains to be investigated. Together, these results suggest that the transcriptional regulation of detoxification gene modules is exceptionally plastic and has been rewired during vertebrate evolution.

The expression of the *PXR* gene in the elephant shark is confined to skin and gills, in clear contrast with what is observed in other species. For example, *PXR* gene transcripts in zebrafish are found in the liver, eye, intestine, brain, heart, and kidney, while in mammals, *PXR* is expressed in the liver, gastrointestinal tract, brain, and retina [23].

The ligand binding profile of the chimaera *PXR* ortholog is also puzzling, with *Cmi*PXR exhibiting a smaller activation for the selected environmental pollutants, in contrast to *PXR* from zebrafish [32]. However, distinct sensitivities towards xenobiotics have been reported across species [32,33,34,35]. Thus, we cannot fully discard the existence of a distinct set of potential PXR-interacting xenobiotics, or other unidentified endogenous ligands, for chimaeras. Nonetheless, our transactivation results, together with the unique expression profile, raise the interesting possibility that *Cmi*PXR could act as a specialized steroid-like sensor in the skin. In fact, previous studies have suggested putative effects of skin-expressed *PXR* in humans and rodents, including the induction of keratinocyte proliferation, immune hyper-responsiveness, modulation of DNA repair mechanisms, and overall skin barrier functions [36,37]. On the other hand, it is known that estrogens participate in skin homeostasis, by modulating collagen deposition, wound healing and scarring, and maintaining skin hydration and elasticity [38]. Furthermore, both *PXR* and estrogen have been directly or indirectly linked to fibrous connective tissue equilibrium [37,39]. Thus, we hypothesize that *PXR* could play a role in estrogen-dependent skin maintenance in chimaeras, contributing to the peculiar appearance of their smooth, rubbery and scale-less skin.

## 4. Material and Methods

### 4.1. Sequence Retrieval and Phylogenetic Analysis

Amino acid sequences were retrieved through blast searches in the publicly-available genome databases, using as reference annotated human VDR, PXR, and CAR sequences. Sequence sampling included major vertebrate lineages: mammals (*Homo sapiens*, *Mus musculus*, *Rattus norvegicus*, *Oryctolagus cuniculus*, *Sus scrofa*, *Bos taurus*), birds (*Gallus gallus*, *Anas platyrhynchos*), reptiles (*Anolis carolinensis*), amphibians (*Xenopus tropicalis*), euteleostei (*Danio rerio*, *Cyprinus carpio*, *Oryzias latipes*, *Oreochromis niloticus*), osteoglossomorpha (*Scleropages formosus*), holostei (*Lepisosteus oculatus*), chondrichthyans (Elasmobranchii: *Leucoraja erinacea*, *Chiloscyllium punctatum*, *Scyliorhinus torazame*, *Rhyncodon typus*; Chimaera: *Callorhinchus milii*), cyclostomes (*Petromyzon marinus*) and invertebrates (*Ciona intestinalis*). The retrieved sequences and corresponding protein accession numbers are listed in the Table S1. A multiple alignment of the retrieved sequences was obtained with MAFFT (multiple alignment using fast Fourier transform) alignment software [40] using default parameters. The final sequence alignment, containing 52 sequences and 659 positions, was used to construct a phylogenetic tree with MrBayes v 3.2.3 (CIPRES, San Diego, CA, USA; http://www.phylo.org/index.php/) sited in the CIPRES Science Gateway V3.3 [41]. The Bayesian analyses were performed under a mixed substitution model with two independent runs of four chains (one cooled and 3 heated) for 1 × 10^6^ generations, and the trees were sampled every 500 generations with a burnin set to 0.25 until the average standard deviation of the split frequencies remained <0.01. The statistical support for each branch is indicated at the nodes and expressed as Bayesian posterior probabilities [42]. FigTree v1.3.1 was used to visualize the tree. Geneious^®^ v7.1.7 (Biomatters Ltd., Auckland, New Zealand) was used to calculate the amino acid identity between human, mouse, zebrafish and elephant shark PXRs.

### 4.2. Synteny Analysis

The genomic region containing the human *PXR*, *CAR*, and *VDR* genes were localized at chromosomes 3 (119.78 Mb), 1 (161.22 Mb), and 12 (47.84 Mb), respectively. The human neighbouring genes, as well as the respective loci (GRCh38.p7), were collected from the GenBank database and used as references to assemble the synteny maps of zebrafish, elephant shark, cloudy catshark, brownbanded bamboo shark, and whale shark. To find the ortholog genes in the genomes of zebrafish (GRCz10), elephant shark (Callorhinchus_milii-6.1.3), and whale shark (ASM164234v2), we performed a BLAST of the human neighbour genes. Four flanking genes from both sides of each target gene were considered against the above-mentioned genomes. In the case of the cloudy catshark (Storazame_v1.0), brownbanded bamboo shark (Cpunctatum_v1.0), and the new version of whale shark (Rtypus_kobe_v1.0), the flanking genes found in elephant shark, as well the previous reference genes of human, were blasted (blast-n: -word_size 10, -outfmt 6, -num_threads 50) against the three recently-built elasmobranchs genomes (https://figshare.com/authors/Phyloinformatics_Lab_in_RIKEN_Kobe/4815111). Importantly, we used the new version of the whale shark genome (Rtypus_kobe_v1.0) to complete the previous syntenic map of the whale shark (ASM164234v2). Next, we manually inspected the BLAST-n results and, using the qstart, qend, sstart, send, and bit score options of outfmt6 format of BLAST software, reconstructed the structure for each gene. To confirm the neighbors’ homology in non-annotated genomes (*C. punctatum*, *S. torazame*, and *R. typus* new version), we used the following strategy: (1) .fasta and .gff files of each genome were used to extract the predicted coding region of each homolog candidate gene (if the blast approach detected the gene fragmentated in different scaffolds, we considered the biggest); (2) we performed reciprocal blast-n (with megablast and dc-megablast algorithm) searches of all candidates genes in the nucleotide database of NCBI (NT-NCBI); (3) if each candidate gene matched against the expected genes (references mentioned above, or ortholog genes in other species), it was kept and used to build the synteny maps.

### 4.3. Construction of Plasmid Vectors

The *PXR* hinge region and ligand binding domain (LBD) were isolated from zebrafish using a polymerase chain reaction (PCR) approach with the specific primers (restriction sites are underlined) F: 5′-atttCTAGAATGAAGAGAGAGCTGATCATGTC-3′ and R: 5′- aattGGTACCCTTTGTGAGGACTTAGGTGTC-3′, and the Phusion Flash master mix (Thermo Fisher Scientific, Waltham, MA, USA), according to the protocol from the supplier. The hinge and LBD of the elephant shark *PXR* was synthesized by IDT—Integrated DNA Technologies, Inc. (https://eu.idtdna.com/pages/home). Both *PXR*s were digested with *Xba*I and *Kpn*I restriction enzymes (NZYtech) and ligated to pBIND (AF264722; Promega) with T4 ligase (Promega, Madison, WI, USA) to produce a GAL4-LBD “chimeric” receptor. The chimeric receptor produces a hybrid protein that contains the Gal4 DNA binding domain (DBD) and acts on an upstream activation sequence (UAS) response element. Plasmid sequences were confirmed using Sanger sequencing (Eurofins GATC, Constance, Germany).

### 4.4. Gene Expression

Total RNA was extracted from the following tissues of elephant shark using Trizol reagent (Gibco BRL): brain, gill, heart, intestine, kidney, liver, muscle, ovary, skin, and testis. One microgram of total RNA from each tissue was reverse-transcribed into cDNA with Superscript II (Invitrogen, Carlsbad, CA, USA) and used as a template for RT-PCR. The following primer pair which spans an intron was used to amplify elephant shark PXR: PXR_F, 5′-TGGAAGATCTCCTGGAAGCACATC-3′ and PXR_R, 5′-GAAGTTACGCTGGAGCTTGTAGTC-3′. Actin was amplified as an internal control to verify the integrity of cDNA using the primers: Actin_F, 5′-GGTATTGTCACCAACTGGGAC-3′ and Actin_R, 5′-AGATGGGCACAGTGTGGGTG-3. The PCR cycles comprised an initial denature step of 95 °C for 30 s, followed by 35 cycles of 95 °C for 10 s, 60 °C for 30 s, and 72 °C for 30 s, and a final elongation step of 72 °C for 5 min.

### 4.5. Transfection and Transactivation Assays

Cell culture and transactivation assays were performed as described in Fonseca et al. 2017 [43]. All ligands used (E2, EE2, TNC and BPA) were purchased from Sigma Aldrich (Sintra, Portugal). All compounds were resuspended in dimethyl sulfoxide (DMSO) at a final concentration of 0.1, 1, and 10 μM; and 5, 50, and 100 μM for BPA. Briefly, Cos-1 cells (Sigma, Sintra, Portugal) were maintained in DMEM (PAN-Biotech, Aidenbach, Bayern, Germany) supplemented with 10% fetal bovine serum (PAN-Biotech, Aidenbach, Bayern, Germany) and 1% penicillin/streptomycin (PAN-Biotech, Aidenbach, Bayern, Germany) at 37 °C with a humidified atmosphere and 5% CO_2_. Cells were seeded in 24-well culture plates, and after 24 h, cells were transfected with 0.5 μg of pBIND constructs (pBIND-CmiPXRLBD or pBIND-DrePXRLBD) and 1 μg of pGL4.31[luc2P/GAL4UAS/Hygro] luciferase reporter vector (DQ487213; Promega, Madison, WI, USA) containing five UAS elements upstream of the firefly luciferase reporter gene, using lipofectamine 2000 reagent (Invitrogen, Carlsbad, CA, USA), in Opti-MEM (Gibco, Carlsbad, CA, USA), according to the manufacturer’s instructions. After 5 h of incubation, the transfection media was replaced with a medium containing the test compounds (E2, EE2; TNC—1, 1, and 10μM; and BPA—5, 50, and 100 μM) dissolved in DMSO (0.1%). Cells were lysed 24 h after transfection, and assayed for Firefly luciferase (reporter pGL4.31) and Renilla luciferase (pBIND) activities with Dual-Luciferase Reporter Assay System (Promega, Madison, WI, USA), according to the manufacturer’s instructions. All transfections were performed with two technical replicates per condition in three independent assays. The results were expressed as a fold-induction resulting from the ratio between luciferase (reporter pGL4.31) and Renilla (internal control for transfection efficiency luminescent activity), and then normalized by the DMSO control. Transactivation data was presented as means of the normalized values (*n* = 3) and bars with standard error of the mean (SEM) from the three separate experiments. The means of the technical replicates were used for statistical analysis with one-way analysis of variance (ANOVA), followed by the Holm–Sidak method in SigmaPlot 11.0 software (Systat Software Inc., San Jose, CA, USA). The level of significance was set to 0.05.

## 5. Conclusions

In this study, we deciphered the early evolution of the central components of the vertebrate “chemical defensome”. Our findings indicate that *PXR* gene orthologs are present in Holocephali but have been probably lost in Elasmobranchii. Moreover, the chimaera *PXR* gene displays a unique pattern of gene expression. Future studies will be required to dissect the molecular wiring of detoxification gene modules in Chondrichthyes.

## Supplementary Materials

Supplementary materials can be found at https://www.mdpi.com/1422-0067/20/9/2331/s1.

## References

[1] Bookout A., Jeong Y., Downes M., Yu R., Evans R.M., Mangelsdorf D. (2006). Anatomical profiling of nuclear receptor expression reveals a hierarchical transcriptional network. Cell.

[2] Capitão A., Lopes-Marques M., Ishii Y., Ruivo R., Fonseca E., Páscoa IJorge R.P., Barbosa M.A.G., Hiromori Y., Miyagi T. (2018). Evolutionary Exploitation of Vertebrate Peroxisome Proliferator-Activated Receptor γ by Organotins. Environ. Sci. Technol..

[3] Santos M., Ruivo R., Capitão A., Fonseca E., Castro L. (2018). Identifying the gaps: Resources and perspectives on the use of nuclear receptor based-assays to improve hazard assessment of emerging contaminants. J. Hazard Mater..

[4] Goldstone J., Hamdoun A., Cole B., Howard-Ashby M., Nebert D., Scally M., Dean M., Epel D., Hahn M.E., Stegeman J.J. (2006). The chemical defensome: Environmental sensing and response genes in the Strongylocentrotus purpuratus genome. Dev. Biol..

[5] Di Masi A., de Marinis E., Ascenzi P., Marino M. (2009). Nuclear receptors *CAR* and *PXR*: Molecular, functional, and biomedical aspects. Mol. Asp. Med..

[6] Zhuo W., Hu L., Lv J., Wang H., Zhou H., Fan L. (2014). Role of pregnane X receptor in chemotherapeutic treatment. Cancer Chemother. Pharmacol..

[7] Tolson A., Wang H. (2010). Regulation of drug-metabolizing enzymes by xenobiotic receptors: *PXR* and *CAR*.. Adv. Drug Deliv. Rev..

[8] Cruzeiro C., Lopes-Marques M., Ruivo R., Rodrigues-Oliveira N., Santos M., Rocha M., Rocha E., Castro L.F. (2016). A mollusk VDR/PXR/CAR-like (NR1J) nuclear receptor provides insight into ancient detoxification mechanisms. Aquat. Toxicol..

[9] Kim D., Kim H., Hwang D., Kim H., Hagiwara A., Lee J., Jeong C.B. (2017). Genome-wide identification of nuclear receptor (NR) genes and the evolutionary significance of the NR1O subfamily in the monogonont rotifer *Brachionus* spp.. Gen. Comp. Endocrinol..

[10] Mathäs M., Burk O., Qiu H., Nusshag C., Gödtel-Armbrust U., Baranyai D., Deng S., Römer K., Nem D., Windshügel B. (2012). Evolutionary history and functional characterization of the amphibian xenosensor CAR. Mol. Endocrinol..

[11] Eide M., Rydbeck H., Tørresen O., Lille-Langøy R., Puntervoll P., Goldstone J., Jakobsen K., Stegeman J., Goksøyr A., Karlsen O. (2018). Independent losses of a xenobiotics receptor across teleost evolution. Sci. Rep..

[12] Kollitz E., Zhang G., Hawkins M., Whitfield G., Reif D., Kullman S. (2015). Molecular cloning, functional characterization, and evolutionary analysis of vitamin D receptors isolated from basal vertebrates. PLoS ONE.

[13] Zhao Y., Zhang K., Giesy J., Hu J. (2015). Families of nuclear receptors in vertebrate models: Characteristic and comparative toxicological perspective. Sci. Rep..

[14] Ravi V., Venkatesh B. (2018). The divergent genomes of teleosts. Annu. Rev. Anim. Biosci..

[15] Bertrand S., Brunet F., Escriva H., Parmentier G., Laudet V., Robinson-Rechavi M. (2004). Evolutionary genomics of nuclear receptors: From twenty-five ancestral genes to derived endocrine systems. Mol. Biol. Evol..

[16] Chimaeras D.D., Carpenter K.E. (2002). The Living Marine Resources of the Western Central Atlantic Volume 1: Introduction, Mollusks, Crustaceans, Hagfishes, Sharks, Batoid Fishes, and Chimaeras [Internet].

[17] Walker T., Stevens J., Braccini J., Daley R., Huveneers C., Irvine S., Bell J., Tovar-Ávila J., Trinnie F., Phillips D. (2008). Rapid Assessment of Sustainability for Ecological Risk of Shark and Other Chondrichthyan Bycatch Species Taken in the Southern and Eastern Scalefish and Shark Fishery. [Internet].

[18] King B., Gillis J., Carlisle H., Dahn R. (2011). A natural deletion of the HoxC cluster in elasmobranch fishes. Science.

[19] Wyffels J., King B., Vincent J., Chen C., Wu C., Polson S. (2014). SkateBase, an elasmobranch genome project and collection of molecular resources for chondrichthyan fishes. F1000Research.

[20] Read T., Petit R., Joseph S., Alam M., Weil M., Ahmad M., Bhimani R., Vuong J.S., Haase C.P., Webb D.H. (2017). Draft sequencing and assembly of the genome of the world’s largest fish, the whale shark: Rhincodon typus Smith 1828. BMC Genom..

[21] Venkatesh B., Lee A., Ravi V., Maurya A., Lian M., Swann J., Ohta Y., Flajnik M.F., Sutoh Y., Kasahara M. (2014). Elephant shark genome provides unique insights into gnathostome evolution. Nature.

[22] Hara Y., Yamaguchi K., Onimaru K., Kadota M., Koyanagi M., Keeley S., Tanaka K., Tatsumi K., Motone F., Kageyama Y. (2018). Shark genomes provide insights into elasmobranch evolution and the origin of vertebrates. Nat. Ecol. Evol..

[23] Bainy A., Kubota A., Goldstone J., Lille-Langøy R., Karchner S., Celander M., Tatsumi K., Tanaka K., Motone F., Kageyama Y. (2013). Functional characterization of a full length pregnane X receptor, expression in vivo, and identification of *PXR* alleles, in zebrafish (*Danio rerio*). Aquat. Toxicol..

[24] Krasowski M., Yasuda K., Hagey L., Schuetz E. (2005). Evolutionary selection across the nuclear hormone receptor superfamily with a focus on the NR1I subfamily (vitamin D, pregnane X, and constitutive androstane receptors). Nucl. Recept..

[25] Goodwin B., Gauthier K., Umetani M., Watson M., Lochansky M., Collins J., Leitersdorf E., Mangelsdorf D.J., Kliewer S.A., Repa J.J. (2003). Identification of bile acid precursors as endogenous ligands for the nuclear xenobiotic pregnane X receptor. Proc. Natl. Acad. Sci. USA.

[26] Kliewer S., Moore J., Wade L., Staudinger J., Watson M., Jones S., McKee D.D., Oliver B.B., Willson T.M., Zetterström R.H. (1998). An orphan nuclear receptor activated by pregnanes defines a novel steroid signaling pathway. Cell.

[27] Krasowski M., Yasuda K., Hagey L., Schuetz E. (2005). Evolution of the pregnane X receptor: Adaptation to cross-species differences in biliary bile salts. Mol. Endocrinol..

[28] Lehmann J., McKee D., Watson M., Willson T., Moore J., Kliewer S. (1998). The human orphan nuclear receptor *PXR* is activated by compounds that regulate *CYP3A4* gene expression and cause drug interactions. J. Clin. Investig..

[29] Moore L., Parks D., Jones S., Bledsoe R., Consler T., Stimmel J., Goodwin B., Liddle C., Blanchard S.G., Willson T.M. (2000). Orphan nuclear receptors constitutive androstane receptor and pregnane X receptor share xenobiotic and steroid ligands. J. Biol. Chem..

[30] Handschin C., Podvinec M., Meyer U. (2000). CXR, a chicken xenobiotic-sensing orphan nuclear receptor, is related to both mammalian pregnane X receptor (*PXR*) and constitutive androstane receptor (*CAR*). Proc. Natl. Acad. Sci. USA.

[31] Moore L.B., Maglich J., McKee D., Wisely B., Willson T., Kliewer S., Lambert M.H., Moore J.T. (2002). Pregnane X receptor (PXR), constitutive androstane receptor (*CAR*), and benzoate X receptor (*BXR*) define three pharmacologically distinct classes of nuclear receptors. Mol. Endocrinol..

[32] Milnes M., Garcia A., Grossman E., Grün F., Shiotsugu J., Tabb M., Kawashima Y., Katsu Y., Watanabe H., Iguchi T. (2008). Activation of steroid and xenobiotic receptor (*SXR*, NR1I2) and its orthologs in laboratory, toxicologic, and genome model species. Environ. Health Perspect..

[33] Lille-Langøy R., Goldstone J., Rusten M., Milnes M., Male R., Stegeman J., Blumberg B., Goksøyr A. (2015). Environmental contaminants activate human and polar bear (Ursus maritimus) pregnane X receptors (*PXR*, NR1I2) differently. Toxicol. Appl. Pharmacol..

[34] Scheer N., Ross J., Kapelyukh Y., Rode A., Wolf C. (2010). In vivo responses of the human and murine pregnane X receptor to dexamethasone in mice. Drug Metab. Dispos..

[35] Sui Y., Ai N., Park S., Rios-Pilier J., Perkins J., Welsh W., Zhou C. (2010). Bisphenol A and its analogues activate human pregnane X receptor. Environ. Health Perspect..

[36] Elentner A., Schmuth M., Yannoutsos N., Eichmann T., Gruber R., Radner F., Hermann M., del Frari B., Dubrac S. (2018). Epidermal Overexpression of Xenobiotic Receptor *PXR* Impairs the Epidermal Barrier and Triggers Th2 Immune Response. J. Investig. Dermatol..

[37] Schmuth M., Moosbrugger-Martinz V., Blunder S., Dubrac S. (2014). Role of *PPAR*, *LXR*, and *PXR* in epidermal homeostasis and inflammation. Biochim. Biophys. Acta.

[38] Shah M., Maibach H. (2001). Estrogen and skin. An overview. Am. J. Clin. Dermatol..

[39] Frazier-Jessen M., Mott F., Witte P., Kovacs E. (1996). Estrogen suppression of connective tissue deposition in a murine model of peritoneal adhesion formation. J. Immunol..

[40] Katoh K., Toh H. (2010). Parallelization of the MAFFT multiple sequence alignment program. Bioinformatics.

[41] Miller M., Schwartz T., Pickett B., He S., Klem E., Scheuermann R., Passarotti M., Kaufman S., O’Leary M.A. (2015). A RESTful API for Access to Phylogenetic Tools via the CIPRES Science Gateway. Evol. Bioinform. Online.

[42] Nascimento F., Reis M., Yang Z. (2017). A biologist’s guide to Bayesian phylogenetic analysis. Nat. Ecol. Evol..

[43] Fonseca E., Ruivo R., Lopes-Marques M., Zhang H., Santos M., Venkatesh B., Castro L.F.C. (2017). *LXRα* and *LXRβ* Nuclear Receptors Evolved in the Common Ancestor of Gnathostomes. Genome Biol. Evol..

